# Principles for websites of the American Medical Association

**DOI:** 10.2196/jmir.2.suppl2.e4

**Published:** 2000-09-13

**Authors:** Margaret Winker

**Affiliations:** ^1^Journal of the American Medical AssociationUSA

## Abstract

The Internet has the potential to provide patients and physicians with rapid access to high quality, timely evidence regarding health and medical diagnosis and treatment. However, many barriers must be surmounted before this potential is achieved. Quality of content must be able to be verified, including the accuracy and timeliness of the information, the source of the information, and the objectivity of the source. Advertising and sponsorship must not influence content and should not be juxtaposed with related content. Individuals must be able to access information without loss of personal privacy. To address these issues, the American Medical Association has developed principles to guide development and posting of Web site content, govern acquisition and posting of online advertising and sponsorship, ensure site visitors' and patients' rights to privacy and confidentiality, and provide effective and secure means of e-commerce. While these guidelines were developed specifically for the AMA Web sites and visitors to these sites, they also may be useful to other providers and users of medical information on the Web.

**Full paper published as:**

Guidelines for medical and health information sites on the Internet: principles governing AMA Web sites by Margaret A. Winker; Annette Flanagin; Bonnie Chi-Lum; John White; Karen Andrews; Robert L. Kennett; Catherine D. DeAngelis; Robert A. Musacchio (JAMA 2000 Mar22/29; 283 (12)::1600-1606). (http://jama.ama-assn.org/issues/v283n12/full/jsc00054.html)

